# On Mental Suggestion, Association, and Reproduction

**Published:** 1862-10

**Authors:** W. G. Davies

**Affiliations:** Chaplain of the Joint Counties Asylum, Abergavenny.


### Art. IV.—ON MENTAL SUGGESTION, ASSOCIATION, AND REPRODUCTION.

By the Rev. W. G. Davies,

(*Chaplain of the Joint Counties Asylum, Abergavenny*).

From Hobbes to the present day considerable attention has been paid by philosophers to this subject. So little, however, was known of its history until recently, that Priestley claims for Locke the honour of having first observed this great mental process, and Hume takes to himself the credit of having first generalized its laws, which, according to him, are resemblance, contiguity in time or place, and cause and effect. Sir James Mackintosh again states that Hobbes was the first to discover the laws of association in anything like their full proportion. Sir W. Hamilton, however, to whom we are indebted for this brief historical sketch, vindicates Aristotle's right, not only to be the founder but the finisher of this doctrine. "The laws of association," he says, "whose evolution modern philosophers fondly arrogated to themselves, are, after these have tried and tired themselves in the attempt, found already developed and applied—I may say, indeed, even generalized into unity—at a single jet, by a single philosopher of antiquity, who for this—but not alone for this—stands the Copernicus and Kepler and Newton of the intellectual world."*

While most modern philosophers have attended to the laws of mental association, some, as it appears to us, have assigned to them undue importance, making the philosophy of the intellectual powers principally, or even entirely to consist of these laws. This, for instance, is done by the sensational school, from Hartley downwards. Take for example, James Mill, Brown, and in the present day, Mr. Alexander Bain, in his able work, *The Senses and the Intellect*. Now, what we have been led to regard as a grave oversight in the system of these philosophers, is the too exclusive examination of the intellect in accordance with the laws of association. Thus, to take the latest of the school: Mr. Bain, in the preface to his work, says that "in treating of the intellect the subdivision into faculties is abandoned. The exposition proceeds entirely on the Laws of Association." What indicates to us the erroneousness of this procedure is not only consciousness itself, which in the last resort is the sole authority on the subject, but even the very laws of association as given by Mr. Bain. Previously, however, to stating our views on this point, it is desirable

* Reid's Works, p. 889.

to attach some more definite meaning than is usually done to the words which appear at the head of this article.

It appears to us that no little confusion attends the exposition of the laws of association from confounding them with those of suggestion. We believe that mental suggestion is more general than mental association, and its result, reproduction. The fruit of our research is contained in the following table:—

*Mental Suggestion.*

| Primary or Production. | Secondary or Reproduction. |
| --- | --- |

In production thoughts suggest others for the first time. Thus like suggests like, and the logical antecedent, its consequent. That is, the suggesting thought, as far as one remembers, has not in one's own mind previously called up the suggested thought.

In reproduction one thought suggests another, because both have previously been presented to the mind as together, or in close succession, and have become associated, and the one recalls the other in consequence of their association. This recalling is rightly named reproduction, secondary suggestion, or with less definiteness, simply suggestion.

Now, the foregoing analysis enables us to see that association implies the prior cognition of the thoughts which are associated. Two objects may be perceived as together, or in close succession, and consequently form a mental coalition, in virtue of which one of the thoughts is afterwards apt to suggest the other. A thought may also, for the first time, suggest another, either like may produce like; or the logical antecedent, its consequent. And such thoughts, if retained, will become associated, so that afterwards one of the thoughts will reproduce the rest. Association, therefore, presupposes the previous knowledge of the thoughts which are blended. Primary suggestion also presupposes the previous knowledge of the suggesting thought. Consequently there must be something to serve as a basis both for suggestion and association, there must be some intellectual power by means of which we acquire, in the one case, the suggesting thought, in the other, the thoughts which are associated together. With these introductory remarks, let us now turn to the Law of Contiguity as stated by Mr. Bain:—

> "Actions, Sensations, and States of Feeling, occurring together or in close succession, tend to grow together, or cohere in such a way that when any one of them is afterwards presented to the mind, the others are apt to be brought up in idea."*

* *The Senses and the Intellect,* p. 318.

What is the precise force of the phrase "presented to the mind" in this statement? We understand it to mean, among other things, perceived. Actions then occurring together, or in close succession, tend to grow together in such a way that when one of them is afterwards perceived or thought of, the others are apt to be brought up in idea. But the condition, 'when one of them is *afterwards* perceived or thought of,' implies that the objects have previously been perceived or thought of as contiguous. One notion reproduces another, because both are associated with each other; and this, as we have just seen, involves the previous acquisition of the notions. The sound of a bell suggests the bell, because the latter has previously been perceived as emitting sound. See hence the error of commencing a classification of the intellectual powers with the secondary or derivative processes, entirely losing sight of the primary processes from which those proceed. Even in cases in which consciousness has only an initiatory part to perform, as for instance, the rhythmical movements of the danseuse, or the execution of the pianist, cases in which the association is between movement and movement, it must at once strike us as evident that the movements must be made, and repeatedly made, before each antecedent action can lead to each consequent action with that admirable precision which is frequently witnessed. So it is with our thoughts: the primary condition of their association is their prior existence as contiguous in perception or in thought; and in proof of this we may point out the fact that, as a rule, one thought reproduces another the more unfailingly, the more frequently it occurs in conjunction with that other. A failure to reproduce on the part of a thought is mostly attributable, either to the infrequency of its conjunction with another thought, or to an unretentive memory, to the want of attention, or to a combination of these causes.

The oversight here pointed out seems to arise from an insufficient analysis of the intellectual operations. It is supposed that objects are in the outset vaguely cognized in the aggregate, and that afterwards the sensations so attained are discriminated into their constituent parts. Perceptions or conceptions,* presented to the mind as wholes, were thought by Brown to suggest other conceptions, and this he called simple suggestion. Two such conceptions known at one time were compared, and thereby their relations ascertained, this he called relative suggestion or judgment. But now it appears to us, after a diligent examination of

* Brown means by conception, the reproduction in imagination of an object of sense as actually perceived. But Sir William Hamilton says, that the word should be limited to what cannot thus be represented in imagination, as the thought suggested by a general term, and that this is the sense in which *conception* has been usually and correctly employed.—*Reid's Works*, p. 360.

consciousness, that this mere cognition of an object in the aggregate without analysis of the same into parts does not exist, except in that mood when the mind is merely passive and unheeding. In active perception which, for all purposes connected with mental science, appears in logical order to be the very first manifestation of thought, there is not this bare apprehension of an object without analysis into parts. And had this fact been recognized, it is very possible that no one would have attempted to make the laws of association primary in their nature. It is not unlikely that here some one will be disposed to remind us of that great field of mind explored by Mr. Bain, comprising sensation and instinct, which are held to be prior to intellect, and to deny that active perception is the very first manifestation of thought. If by sensation is meant a knowing operation, then we cannot see, as will appear further on, what occasion there is to make a distinction between it and perception. As for instinct, it is either a reflex action consequent upon a mere impulse received from passive perception, or it is an act of implicit thought, thought manifest only in its results, and consequently has to be discussed along with the more advanced operations of the intellect, as for instance, those to which we owe self-evident truths so called.

Indeed, we cannot help thinking that mental philosophy, take which system we will, stands, as regards the account which it gives of the origin of our knowledge, in decided need of rectification. Let us select, for instance, the Scoto-Kantian system. Mr. Mansel, in his able and learned work, *The Prolegomena Logica,* renders the following account of the origin of our notions :—

> " In mere intuition, all that is simultaneously presented to the sense appears as one whole; but mere intuition does not distinguish its several parts from each other under this or that notion. I may see at once, in a single panorama, a ship upon the sea, an island behind it, and the sky above it. To mere intuition this is presented only in confusion, as a single object. To distinguish its constituent portions, as sea and island, ship and sky, requires a comparison and a classification of them relatively to so many separate concepts existing in the mind, and such classification is an act of thought."*

Now, what we contend for is, that if intuition, or what we suppose can be included under it, sensation, be an act of knowing, it differs nothing from perception ; for, as far as we can discover, there is no more fundamental knowing power than that. To make a distinction, therefore, between sensation and intuition, on the one hand, and perception on the other, is to violate the maxim : *entia non sunt multiplicanda præter necessitatem.* It is possible,

---

we repeat, that the perceptive faculty may be in a passive, drowsy, unheeding state, a state in which it does not exert itself to analyse its objects; but whenever it is in an active mood, whenever it scrutinizes its object, it then in a series of acts of attention discriminates the whole into its parts, and gives expression to each act in a proposition or asserting sentence. While, however, we contend that, considered as a knowing act, sensation is one with perception, we are well aware that there is a sense in which the word sensation must stand for the thing known. Thus we say bodily pain is a sensation; but the sensation, the pain, does not know itself, or is not mere knowledge, it is cognized by perception. The sensation, the pain, is perceived.

1. After a long and conscientious examination of the human intellect, we have been brought to see that there are three faculties, from one or other of which all knowledge takes its start. These are Perception, Conception, and Reason. The first in logical order of these is Perception. Before we acquire knowledge by means of this faculty internally or externally directed, the mind has merely a potential existence. Mind only exists for us in consciousness, and then it first becomes clearly conscious when it perceives an object, which object, in the first place, must be ourselves as possessed of extended animation. We do not mean to assert that Perception operates actively at once, for it has to be educated into action. First it manifests itself passively, then, as it acquires strength, it becomes capable of making discriminating efforts, of analysing its object, and becoming intimate with it. Its first object, we have stated, is ourselves as possessed of extended animation. Thus it is that we realize the basis of self or the *ego*. We subsequently by means of the senses experience what in contrast to the *ego* is called the *non-ego*, the external world. We must however desist from entering into this branch of our inquiry in this place, and limit our remarks to what is absolutely necessary for the clear exposition of the laws of association. For a fuller explanation of the branch here alluded to we are reluctantly compelled to refer the reader to another source.* We must in this place then content ourselves with simply stating the law of Perception, the origin, as we believe, of the first law of association:—

> Perception is the cognition of an object as a whole (that is, whatever can form the subject of a proposition or asserting sentence) by the discrimination, 1, of the parts as severally different from the whole; 2, of the parts as different from each other: 3, of the whole as different from other wholes.

An act of perception is expressed by the proposition. The subject of the proposition, which must invariably be a whole

* *The A, B, C, of Thought, &c.* Williams and Norgate.

term, represents the whole, which is that of comprehension ; and the predicate represents a part of this whole. But as we cannot at once attend to all the parts of a whole, but have to do so in detail, so we can only have one thing asserted of a subject in a simple proposition. Those propositions in which several attributes are predicated in succession of a single subject are, we need not say, so many propositions in an abbreviated form as there are attributes expressed. Thus, the weather is wet and cold, is merely a concise form of affirming that the weather is wet, and the weather is cold.

The above being the law according to which we perceive objects, is also the source of the fundamental law in agreement with which one thought reproduces another. The law of contiguity is a statement, that thoughts are apt to recall each other in the order in which they originally entered the mind. This law, under the name of redintegration, is stated by Sir W. Hamilton as follows:—"Thoughts once coidentical in time are, however different as mental modes,* again suggestive of each other,. and that in the mutual order which they originally held."† This law is evidently derived from the law of Perception. A whole being cognized by an analysis of its parts, the presentation of any of these parts to the mind tends to call up the whole to which they belong. We perceive objects together or in close succession. If we behold a wide landscape, the thought afterwards of some part of this landscape, or of some event connected with it, will tend to recall the thought of the whole, or series of wholes which originally were present to the mind. Contiguity of objects in place, or in time, is resolvable into the yet more general law of contiguity in perception. Things perceived together, or as immediately following each other, are apt to suggest each other in the same order. We can discover no more fundamental law of *actual* association than this. There is indeed a more general law, but it is one of possible and not of actual association, and therefore of very vague application. This is the law of associability :—

> All thoughts of one and the same mind are associable, or capable of suggesting each other.

2. The second law of association is the law of similarity. This is stated by Mr. Bain in the following words :—

> "Present actions, sensations, thoughts, or emotions, tend to revive their like among previous impressions."‡

Now, in view of this law we have again to ask, whence springs the notion of likeness ? We must be able to apprehend simi-

---

* Stars and night are different mental modes. The appearance of the full moon now, and its appearance on former occasions are similar mental modes.

† *Reid's Works*, p. 913. ‡ *The Senses and the Intellect*, p. 451.

larity before we can say that two objects are alike, and before one object will suggest another which is similar to it. Here again note an error resembling that which we had to expose in regard to the law of contiguity, namely, the making that an original law which is simply a derivative one. Before we can be brought to think of a man by another whose likeness reminds us of him, we must have something in us which is conscious of resemblance among objects. This office we assign to the second originating faculty, Conception, the law of which we conclude to be the following:—

Conception is that faculty which cognizes resemblance between two or more wholes as cognized by Perception.

Conception presupposes Perception, for the objects between which it apprehends resemblance are supposed to be already known as regards other attributes by Perception. Resemblance is a relation between things, and before the things related can be known as such, they must be known in respect to that which is the basis of the relation. If you conceive this face to be like that face, both, irrespective of their likeness to each other, must previously be cognized by Perception as faces. For this reason we call Conception the second originating faculty or power.

Conception is expressed by the proposition having the predicate, or both subject and predicate, common to a plurality of individuals. When we conceive, we are conscious that A and B have a similar attribute, C. Perception is conscious that A is $C'$, and that B is $C''$, but it detects no resemblance between $C'$ and $C''$. This is done by Conception, which enables us therefore to represent both $C'$ and $C''$ by one term C, which is then a common term, common to both A and B. Without Conception we should have no common words, no classification. Now to come to the subject of this inquiry, the law of similarity is clearly derived from the law of Conception. Like suggests like, because we are possessed of a faculty which apprehends resemblance among objects. Nothing seems to us more evident than this.

A repeated perception of one and the same object heightens the adhesiveness with which that object is retained by the mind. Now it has been a subject of debate whether an object that has been frequently presented to the mind could be recognized at each time of presentation as an identical object, were it not that by the law of similarity it was known to be like itself whenever presented. Without the principle of similarity there would be no memory, no remembrance when a thought springs up in the mind that it had been there before. The thought of this moment would not recall the remembrance of similar or identical thoughts in the past. To this it may possibly be objected, that Conception takes note of resemblance not of identity, that resemblance, being a relation between two wholes, involves plurality, that an identical object non-identically presented is not regarded as several objects resembling each other, but as one and the same object, and cannot therefore be cognized as such by Conception. By the law of contiguity each fresh presentation of an object recalls the thought of the past presentations of the same, and both form a complex whole. The object of this moment drags after it, as it were, the remembrance of a long thread of past presentations of that object, this thread being considered a part of the whole experience. And now, bear in mind that this suggested train is not of similar objects, but of an identical object, non-identically presented.—This we doubt, for in opposition to this view the question arises, what link beyond that of contiguity, that by which different mental modes are united, can there be between this moment's consciousness and the remembrance of that of preceding moments, except their identity were recognized by some means or other? One at this moment is conscious of A, but it is the third time of its presentation, and it suggests $A^2$, which suggests $A^1$. Now here is $A^3$ associated with $A^2$, and that with $A^1$, according to the law of contiguity, but they, for all that Perception declares to the contrary, are not recognized as identical, but may be A, B, and C, three different things. It is manifestly to Conception, therefore, that we must look for the notion of identity. If two similar things are known at the same time, or in close succession, they are known to be similar; but if one and the same thing be known at various times it is looked upon as identical. Similarity then involves plurality of objects: identity simply involves plurality of presentation in relation to a single object. We should not say that the pen in this hand is identical with the pen in the same hand, for there is no room for comparison. There must be non-identity of time, in order to give any significance to the notion of identity. Thus there would be some sense in saying that this pen is the one we had yesterday. What will show the correctness of this view is, we think, the following illustration:—Tom passes every morning by a gate which has a large ball on each pillar. Whenever he sees one of the balls, the suggestion occurs to his mind that it is the one he has seen every morning on which he has passed by the gate; and this suggestion is morally certain to be correct. But let us suppose, for the sake of illustration, that one ball was frequently substituted for another, each ball being a facsimile of every other. In this case the suggestion of identity would be at fault; and if Tom knew that one ball was changed for another, resemblance would be suggested to his mind, and not identity. This proves that the notion of identity and of resemblance are nearly akin to each other. They both involve an act of Conception.

From the foregoing discussion, it seems that without Conception we should have no notion of identity, and that, therefore, the past would be complete oblivion to us. Thoughts, the offspring of past perception, might start up in the mind, but they would be quite strange to us, because we could neither remember their identity, nor their similarity to previous thoughts. We consequently see how intimately blended in their operations are the two faculties with which we have been concerned.

It has been maintained by some that the law of contiguity contains the law of similarity. Compare both laws together, and you detect in this respect a sameness between them, namely, that they are both expressed by the proposition, and therefore come under the law of whole and part, or subject and predicate. A part of a whole is cognized both by Perception and Conception, they consequently in this respect come under the same law. This shows that generically they are similar. Specifically, however, they are so dissimilar that Perception can never yield the notions due to Conception, nor this yield the notions due to the former faculty. Perception is the source of division in the world of mind: Conception of unity. Perception delights in the many: Conception in the one. Perception discriminates, distinguishes, analyses, divides, enters into minutiæ, examines individuals: Conception classifies, reduces facts to law, revels in analogies; it simplifies our knowledge by reducing it to order under a few leading heads, and obviates the necessity of giving every object a proper name by making common words perform all the needs of thought; more especially Conception takes note of that thread of identity which enables us to connect the present with the past. It thus appears that these two faculties have quite separate spheres of action, and that you cannot bring them wholly under the same law. The relation between them may be thus set forth. In the first place: Two faces have a character of their own, which fact is known by Perception. But in the second place: The two faces in the character which they possess resemble each other, which fact is known by Conception. From this we see that Conception does not come under the law of whole and part co-ordinately with Perception. Indeed if it did, there would be no reason why it should be called a separate faculty. It comes under the said law then based upon Perception. The law of subject and predicate is the fundamental law of thought, under it must be ranged every intellectual operation, but as a species, possessed of that characteristic which distinguishes it from every other operation.

3. We now approach another phase of this question, and one perhaps which has been more debated than any other, namely, the origin of necessary and universal truths, and of the notion of causation. These are accounted for by the sensational school by association. Uniformity of co-existence between two facts begets so strong a coalition between them, that their co-existence is looked upon as necessary. And uniformity of antecedence and consequence between two facts begets a like association, and generates the notion of a cause, or necessary antecedent. The necessity, it is said, is simply a psychological one. By a fundamental law of mind two facts which are invariably met together become associated, and this association becomes so strong that it cannot be undone. From the inseparability of thoughts, however, it is contended that we cannot justly infer the inseparability of the things from which they are derived, or to which they refer. Our forefathers, from the indissoluble nature of their associations, could not bring themselves to credit the existence of antipodes, or the truth of the Copernican theory, while we who have been brought up to receive different associations on these points wonder at the inaptitude of our ancestors. But let Mr. J. S. Mill, the great upholder of this theory, speak for himself:—

"There is no more generally acknowledged fact in human nature, than the extreme difficulty at first felt in conceiving anything as possible, which is in contradiction to long established and familiar experience, or even to old familiar habits of thought. And this difficulty is a necessary result of the fundamental laws of the human mind. When we have often seen and thought of two things together, and have never in any one instance either seen or thought of them separately, there is by the primary law of association an increasing difficulty, which may in the end become insuperable, of conceiving the two things apart. This is most of all conspicuous in uneducated persons, who are in general utterly unable to separate any two ideas which have once become firmly associated in their minds; and if persons of cultivated intellect have any advantage on the point, it is only because having seen and heard and read more, and being more accustomed to exercise their imagination, they have experienced their sensations and thoughts in more varied combinations; and have been prevented from forming many inseparable associations. But this advantage has necessarily its limits. The most practised intellect is not exempt from the universal laws of our perceptive faculty. If daily experience presents to anyone for a long period two facts in combination, and if he is not led during that period either by accident or by his voluntary mental operations to think of them apart, he will probably in time become incapable of doing so even by the strongest effort; and the supposition that the two facts can be separated in nature, will at last present itself to his mind with all the characters of an inconceivable phenomenon."*

Mr. Mill, in the ablest and strongest manner, here states the view to which we are opposed, but he does not convince us that this is the true explanation of the notion of necessity, or rather

---

* *System of Logic*, pp. 265-6.

of necessary conjunction among facts, for this notion is quite independent of habit or of long-continued association. That twice two are four necessarily and universally, is as evident the first time that the truth is realized, as at the millionth or billionth time. According to Mr. Mill's explanation we owe all "so-called" necessary and universal truths to the law of similarity, or to Conception. But this faculty completes its office when it takes note of identity and non-identity, resemblance and non-resemblance, when it acquires common terms, and realizes classification. There is nothing like the notion of necessary conjunction within its category. In proof of this we may refer to those uniformities which are unbroken to Conception, and yet are not thought to be necessary and universal, for instance, such statements as, "young grass is always green," "all snow is white." Since we see, and hear, and read, that all snow is white, why do we not look upon it as necessarily so? We have now been acquainted with Mr. Mill's argument on this point, say, for the last ten years, yet during all that period, although we have known how prone the mind is to be tyrannized over by its associations, and how necessary it is to bring them under the control of reason, we have never been able to think that twice two only contingently make four, or that fire, some time or other, may not burn. Consequently what we mean to assert is, that it is not the firmness of the association that prevents our doing this, for if this had been the obstacle, we feel confident that we should have been able to surmount it. No, the obstacle is the absence of any other alternative. We do not regard grass as necessarily green, because we are not reduced to the necessity of thinking this and nothing else. We are, however, constrained to think that two intersecting straight lines will never meet, and that all ice is cold, because there is no other alternative. The faculty which enables us to detect necessary conjunction, we call Reason. Perception tells us that two facts are conjoined, Conception that they are uniformly conjoined, but it is Reason, as a conclusion drawn from certain premisses, which declares to us that the facts are necessarily so joined. The law of Reason we consider to be the following:—

Reason* is that faculty which by comparing together two propositions bearing a certain relation to each other, becomes cognizant of a third proposition.

The principal example of this law is the following, to which, as it appears to us, we owe the origin of necessary truths or conjunctions:—

---

* We do not observe the distinction which has been made between reason and reasoning. By reasoning we mean the operation of reason. We can find no evidence that we owe the origin of axioms, or first principles, to Reason, or νοῦς, as an intuitive or non-inferring faculty.

If the existence of any circumstance is attended with the existence of another circumstance,

And the non-existence of the first circumstance is attended with the non-existence of the second,

Then the existence of the first circumstance is necessary to the existence of the second.

This form of reasoning we call the Canon of Induction. To it, as we have endeavoured to indicate elsewhere,* we owe the origin of all self-evident truths so-called, or all first principles.

Now what Reason has to do with association may be described in one phrase, the reproduction of logical results. We think it almost certain that each faculty must retain its own knowledge. Perception does not remember the knowledge acquired by Conception, nor does that faculty remember the knowledge acquired by Reason, but each seems to retain its own. If this be true, Reason can alone be made to reproduce a former inference, and Conception alone, to reproduce the notion of resemblance. In the case of Reason, however, it must be borne in mind, that being the third originating faculty, the relations which it detects are relations among facts which are known by the two prior faculties. Thus in the case of causation, that an antecedent event is followed by a consequent event is cognized by Perception, that this event is invariably followed by that event is observed by Conception, but that the antecedent event is necessary to the existence of the consequent is inferred by Reason in accordance with the canon of induction. Now in the reproduction of the knowledge of these facts, we conclude that each faculty is strictly concerned with its own. To think otherwise will be tantamount to holding that either faculty can act for the other, or one for all, but of this we have no evidence, although in deference to the opinion of those who maintain the unity of the mind we have carefully sought it. We also maintain the unity of the mind, but like that of the whole man, it contains much diversity in the unity. Man has but one mind, as he has but one body. As, however, in the organism there are a series of systems, the first in order being the necessary antecedent of the second, and that of the third, so we discover that it is in the mental nature of man. We find, for instance, that Reason is the third originating faculty. The grounds which we have for allotting this order to it are these :—It involves Perception, in so far as it involves for its premisses two propositions at least. This is very evident, but it is not so evident that it presupposes Conception; that it does however is easily indicated. We have stated above that memory would be impossible without Conception. In fact, a mind without this faculty would be completely imbecile. In order to reason, it is necessary that we retain the premisses while we draw the conclusion, but this we

---

* *The A, B, C of Thought, &c.*

should be totally unable to do unless we were conscious of this moment's thought in conjunction with the persistency of that of immediately preceding moments as identical with it. In short, thought would be impossible without Conception to connect one notion with another by a thread of identity. This, then, is one cause why we call Reason the third in order of the originating faculties. Another obvious cause is, that the premisses from which we draw a conclusion are almost invariably general propositions, the offspring of Conception. And if the conclusion be a necessary and universal proposition, it must be equally clear that Conception must co-operate with Reason in order to obtain it. For Reason like Perception is conversant solely with the singular or the individual. All general, and *à fortiori* all universal truths involve the operation of the conceptive faculty.

In a train of thought, ideas do not merely occur in a chronological, but in a causal sequence. Given a part, it causes the whole to which it pertains to start into consciousness. It seems, however, to us that the notions which enable us to have a train of thought passing through the mind are mostly those parts of a whole which constitute relation to other wholes. Contiguity in perception leads us to think of those things with which a whole was coadjacent, and these perhaps are indicated by the following parts of speech, adjectives in the comparative and superlative degree, verbs transitive and passive, and prepositions. Tom is *taller* than James, who *beat* John in a fight which he had with him *in* the playground just *before* the commencement of last Christmas holidays. The words in italics are the parts of speech enumerated above. Do they not serve as connecting links of thought? Then again Conception suggests the relation of likeness, and of unlikeness, which exists among objects, while Reason suggests the logical relations which subsist between them, as expressed by the illative conjunctions.

But perhaps by far the most important point in connexion with reproduction and trains of thought, is the method which is necessarily involved in the acquisition of knowledge. The objects which are most repeatedly brought before the mind are, as the rule, most easily retained, and these necessarily form the basis, the rudiments, or the simplest portion of the knowledge of any subject. In proportion as facts cease to be fundamental, they become less general, and are then not so likely to be retained. The skeleton of our knowledge therefore is most likely to be firmly fixed in the mind, and most apt to be reproduced. This is consonant with the method of nature, in which we first have the foundation, then the superstructure; first the frame-work, then the filling-up; first the more simple and general, then the more complex and peculiar.

4. The erroneousness of commencing a classification of the intellectual powers with the laws of association is, we believe, most signally evidenced in the attempt which Mr. Bain makes to explain, in accordance with these laws, the origin of the notion of extension. "I hold, as regards extension in general," says Mr. Bain, "that this is a feeling derived *in the first instance from the locomotive or moving organs ;* that a definite amount of movement of these comes to be associated with the sweep and adjustment and other effects of the eye ; and that the notion when full grown is a compound of locomotion, touch, and vision, the one implying and recalling the others."* Then, again, he affirms that, "The conclusion therefore is, that extension, size, or magnitude, owes not *only its origin* but its essential import or meaning to a combination of different effects associated together under the principle we are now considering (that of contiguity). Extension, or space, as a quality, *has no other origin,* and no other meaning than the association of these different sensitive and motor effects."† It seems to us that these passages are quite inconsistent with each other. We are told that extension owes its origin to association under the law of contiguity, and we are also told that it is a feeling derived in the first instance from the locomotive organs. Now we decidedly prefer the latter statement to the preceding one, because, as it seems to us, it very rightly assigns some other origin than association to the notion of extension. Association, whether it be confounded with primary suggestion or not, involves previous knowledge. But if, as we think should be done, we divide it from primary suggestion, it then involves the prior existence of the wedded thoughts, which therefore cannot be originated by it, and to maintain this is to commit a flagrant *petitio principii.* But perhaps all that Mr. Bain means to assert, though he does not do so, is, that locomotion, touch, and vision contribute the sensations (we say the perceptions) out of which the full-grown notion of extension is formed, and that what one of these supplies recalls the contributions of the rest. Thus sight conjures up the notions derived from touch and locomotion, and that so promptly and unfailingly that we seem to behold distance by the eye alone. This interpretation, however, is inconsistent with the view which attributes the origin of the notion of space to association. Besides, although Mr. Bain's views may be understood to signify that sensations thus precede their coalition, yet we must remind the reader that sensations or intuitions are commonly held, and held by Mr. Bain, to be prior to thought, or the exercise of intellect, and that therefore, according to him, any really intellectual realization of space must indeed commence

* Pp. 367, 8. † P. 368.

with association. This amounts to saying that certain sensations or intuitions when apart from each other afford no knowledge of dimension in any sense, but that as soon as they form a coalition the notion of extension begins to be entertained. This theory, we cannot help thinking, is as if some one were to hold that we could not see any part of St. Paul's, till we had seen the whole of it.

We are also at issue with Mr. Bain as to the origin, which, contrary to his principles, he is obliged to claim for the notion of extension. He says that it is derived in the first instance from the locomotive or moving organs. We have every reason to believe that its basis must be sought for in the very lowest depth of our nature. We trace it to the fundamental sense, which gives us the perception of extended animation. We perceive the life of one part of the body as locally out of the life of another part, and the peculiarity of this perception is, that it remains steadfast during our waking moments, never intermitting. For being the basis of all manifestations of consciousness, if it subside all other manifestations must do the same. If the heart ceases to beat, life becomes extinct, and so if the perception of extended life becomes dormant, consciousness ceases to disclose itself. We believe therefore that we derive our rudimentary knowledge of dimension from the extended animation. This is the view taken by Sir William Hamilton. He does not however attach to extended life, as the fundamental object of which consciousness must never cease to be aware, or be dormant, the same importance that we do. We make it the irremovable base of the conscious man. After the fundamental sense, the next source of our knowledge of dimension seems to be touch, which complements the former sense. Touch reveals to us the organic surface with something externally in contact with it. This something is extended commensurately with the extension of the animation in that part of the organic surface which is revealed to us in the same act of contact. That is, such surface then only is cognized, when something external is known as being in contact with it, resisting it, and extended commensurately with its own extension. The leading fact, then, which touch discloses, is objective dimension, namely, that of our organism as the material seat of the pervading animation, and also that of the material surface which is in contact with our organism. Objective dimension therefore appears to be made known, in the first place, by means of the fundamental sense which is cognizant of the extended animation that spreads throughout the corporeal man, the measure of exterior dimension in touch being the exended life of the part in which contact with an external object takes place.

But it is necessary that the motive power should come to the aid of touch, before the latter becomes possessed of its full power. Instead then of making the motive power the very origin of the notion of extension, as Mr. Bain does, we find every reason for believing that it comes third in order. First, the fundamental sense, then touch, then the muscular sense. The fundamental sense originates the notion of extension*; touch and the muscular sense in union with vision complement it. Without the fundamental sense, not only would there be no notion of extension, but no consciousness, and therefore no touch and muscular discrimination. In order, however, to elucidate this fact more fully, it becomes necessary to have recourse to the following analysis :—

Let us regard the human body,

1. As possessed of extended life.
2. As receiving pressure from an external body.
3. As having either of its limbs moved by an external agent.

Now the pressure and the movement here spoken of are entirely independent of the motive power. When the motive power is exerted :—

1. To press an external body, the sense of pressure is the same as above (2), but there is added a consciousness of motive force exerted.

2. To move the hand, for example, in order to handle an object, the sense of pressure is the same as above (2), the sense of movement is also the same as above (3), but there is added a consciousness of motive power as exerted to move the limb in order to press the object.

Now in this analysis we have a decided line drawn between the function of touch and that of the muscular sense. The latter presupposes the former. There can be no outflow of motive force in order to handle an object without the facts apprehended by touch as described above. But these facts can be cognized when the motive power is at rest.

Seeing that there is this difference between touch and the muscular sense, let us ask what share each of these has in the perception of the external world? Touch is conscious of the *non-ego* as an extended object, offering resistance to, or exerting pressure upon, the cutaneous surface of the body. The muscular sense is also cognizant of resistance, but it is in every case of resistance to the motive power, which nevertheless cannot act without the co-operation of touch. For touch may be operative when the muscular sense, would yield us a very inadequate knowledge of the tangible world. The muscular sense, by means of the sweep of the arms, and the movement of the legs, enables us to perceive direction and distance, and consequently greatly increases our knowledge of space. Figure, too, is at first chiefly cognized by the muscular sense attending to the direction in which the hand moves when handling an object. When to these senses sight is added, our notion of extension in all its aspects of length, breadth, and thickness, becomes full grown. Much could here be said of the phenomena of vision, showing how by association with touch and the muscular sense sight becomes as it were a superior sense of touch, making us acquainted with remote objects, and with all the varieties of figure, but we must draw to a close, and continue to remark that we owe the origin of our knowledge of extension exclusively to the fundamental sense. The consciousness of bodily movement presupposes the consciousness, as an extended object, of the limb which moves. This being the case we cannot of course hold that the locomotive energy is the origin of extension. We could not be conscious that we moved a limb unless by means of the fundamental sense complemented by touch, we were already conscious of that limb as a portion of the corporeal *ego*. The muscular sense therefore enables us to realize space by means of the movement of limbs which are already perceived by the fundamental sense and by touch to be parts of the extended *ego*, or corporeal man.

Now is this not the conclusion to which comparative physiology leads ? First in the series of animated beings we have those which are devoid of locomotive power. Yet each of these, in so far as it is an animated being, seems to possess, though not in the same endowment as the higher animals, the fundamental sense, together with the sense of touch and that of taste ; and in consequence to be able to realize both its own existence and that of something external to itself.

The relation which exists between the fundamental sense and touch seems to be this: The former operates in two directions, in the first place, internally, in the second place, externally. When operating in an internal direction it is, during our waking moments, non-intermittent, and forms the basis of every other manifestation of conscious life, as also, in conjunction with conception, of our conscious unity, or personal identity. When operating in an external direction it seems to constitute the sense of touch. Its inward operation forms the condition of its outward operation.

What recommends for adoption the view which we have taken in this article of the laws of association, is not only its presumed faithfulness to consciousness, the only ultimate authority in the matter, but also the avoidance which it seems to ensure of that hazy and confused view which must arise from confounding primary suggestion with secondary, and original with derivative laws. We state in the first place the origin of our knowledge, we then state the laws of its reproduction, and show that these are respectively derived from the laws of Perception, Conception, and Reason. We have, however, only been able to add a few thoughts on the principles of this important subject, hoping tha they will be found useful as a basis for further inquiry. And here, before concluding, we must add that although we have been under the necessity of differing from Mr. Bain as to the origin of our knowledge, we consider his work the most advanced that has as yet been given to the world on the laws of association. It forms a treasury so full that there is little occasion for any one to go elsewhere in search of information on this subject.

---

